# Assessment of radiological parameters and metal contents in soil and stone samples from Harrat Al Madinah, Saudi Arabia

**DOI:** 10.1016/j.mex.2018.05.008

**Published:** 2018-05-22

**Authors:** Saleh Alashrah, Atef El-Taher, Howaida Mansour

**Affiliations:** a Physics Department, College of Science, Qassim University, Buraydah, 51452, Saudi Arabia; b Physics Department, College of Science, Al-Azhar University, Assuit, 71452, Egypt; c Physics Department, College of Science and Art, Ar Rass, Qassim University, Saudi Arabia; d Physics Department, Faculty of Women for Arts, Science and Education, Ain Shams University, Egypt

**Keywords:** Natural radioactivity, Radiation hazards, Annual effective dose rate

## Abstract

The current work deals with measurement and distribution of natural radionuclides for twelve (12) soil and fifteen (15) stone samples collected from Harrats Al Madinah in western region of Saudi Arabia. Two methods were used in this investigation gamma-ray spectrometer (GRS) and X-ray fluorescence (XRF).The activity concentrations of radionuclides (^226^Ra, ^232^Th and ^40^K) were measured using γ-ray spectrometer NaI(Tl) model (A320) made in the U.S.A. The average values of the concentrations of ^226^Ra, ^232^Th and ^40^K were 37.5 ± 0.1, 28.0 ± 0.5 and 300.6 ± 1.7 Bq/kg respectively. The obtained results show that the mean radium equivalent activity, annual effective dose, external and internal hazard indices and radiation level index were 100.67 BqKg^−1^, 55.63μSv, 0.27, 0.37 and 0.73 respectively. The results were compared with the recommended limits in the literature from other locations and with the global allowable limits recommended by International Commission on Radiological Protection and United Nations Scientific Committee on the Effects of Atomic Radiation (UNSCEAR). The obtained results are concordant with the magnitude of safe criteria and exposure risks which were recommended in public papers. The current study is considered as the first baseline data for the natural radioactivity and metal contents measured by X-ray fluorescence method in the Harrat Al Madinah city.

## Graphical abstract

**Figure:**
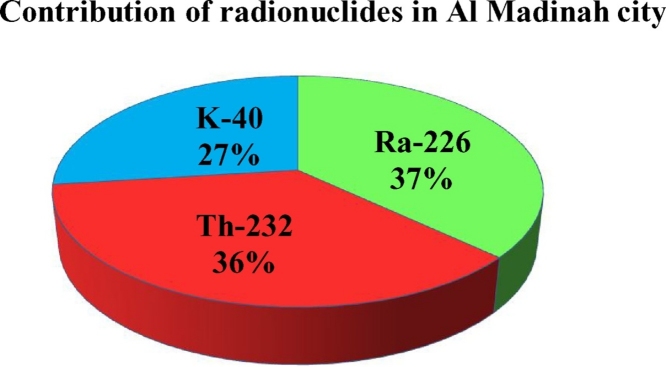

## Method details

Natural background radiation are the main sources of outdoor terrestrial gamma dose as humans are continuously exposed to ionizing radiation from natural radionuclides like ^226^Ra, ^232^Th and ^40^K from the soil. [1] A person is exposed approximately eighty percent of the total radiation dose in a year [2,3]. Soil is one of the most common sources of natural radionuclides. The activity concentrations of radioelements and chemical elements depend on the geological setting and geochemical properties of each region caused by surrounding environment. Thus, the information of the contents of radionuclides is necessary to estimate the radiation risk on environment [[4], [5], [6], [7], [8], [9]].

Al Madinah El Monawara is one of the most important cities in Saudi Arabia where people visit every year from all over the world. There is a lack of data about the contents of natural radioactivity on the studied area. This research is considered the first study in that region. However, a continuous monitoring and assessment of radionuclides fingerprints and contamination is advocate.

The current study focuses on radiometric and chemical analysis of soil and stone samples collected from Al Madinah city in Saudi Arabia using X-ray fluorescence (XRF) and NaI (Tl) scintillation detector.

## Geological setting

**Fig. 1:**
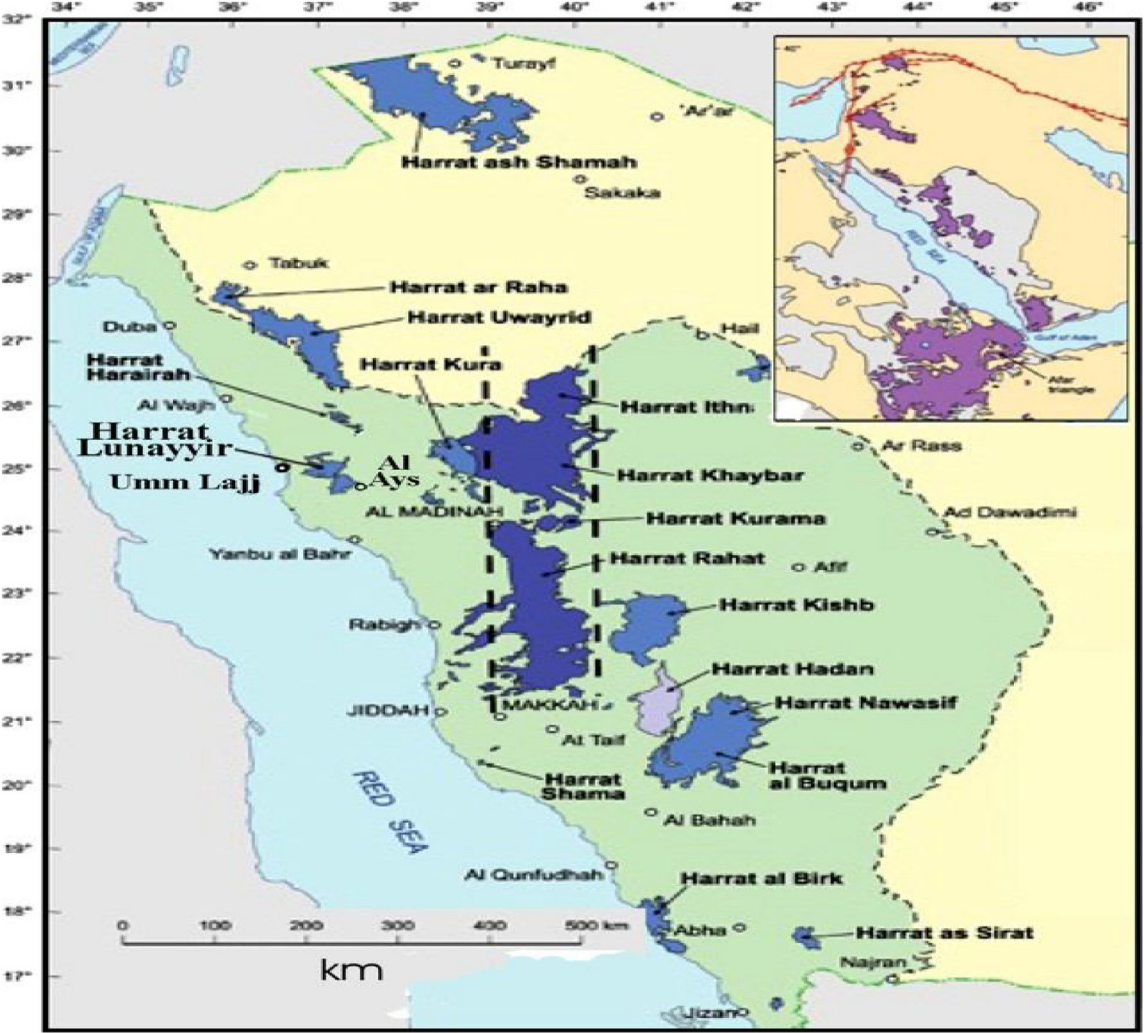
Location of studied area.

The study area lies between longitudes 34° to 46° and latitudes 17° to 32° in the western region of Saudi Arabia Fig. 1. The most important characteristic of Harrat Al Madinah from the geological point of view is the existence of volcanic eruptions. The soil and stones found in the area are dark basaltic rocks formed by the eruption of lava from the ground to the surface [10].

## Experimental technique

### Samples preparation

About 0.5–1.0 kg of twelve (12) soil and fifteen (15) stone samples were collected between 0 and 10 cm of land surface from different locations in Al Madinah, KSA. There are many steps to samples preparation before radiometric and chemical analysis as follows:

(1) Soil and stone samples were dried at 105 °C to remove moisture completely, and then split by quartering to ensure the distribution of the elemental contents.
(2) Crushed and sieved through a 200 mesh to become homogenous.
(3) For radiometric analysis, each sample was weighed and placed in a 350 cm^3^ beaker, and then sealed tightly for four (4) weeks to allow for secular equilibrium to ensure that radon gas is confined within the volume in the sample [11].
(4) For X-ray fluorescence (XRF), about 8 g from powder sample and 1.6 g of wax were taken and pressed under suitable pressure to prepare discs for elemental measurements [[12], [13], [14]].

## Instrumentation and calibration

A gamma ray scintillation spectrometry NaI(Tl) detector model A320 and SN A3200829 was used to determine activity concentrations of radionuclides. The hermetically sealed assembly is coupled to a personal computer-multichannel analyzer (Canberra AccuSpec) model MCA2500R and serial 25,066. The detector was shielded to reduce background radiation using lead shield (100 mm thick) and copper shield (0.3 mm thick). Quantum Gold version 4.04.4 PGT (Princeton Gamma- Tech) was used to analyze gamma ray spectrum [4]. An empty beaker was used in the same condition of samples measuring to estimate the background radiation around the work environment. The accumulated spectrum of background was subtracted from specified photo-peak energy of each sample to get accurate measured activity.

## Calculation of activity

The measured activity in (Bq/Kg) for soil environmental samples was calculated using the following equation:-

(1) $$A(Bq/Kg)=.\left[\frac{1}{\eta }\left(N_{E}-.N_{B}\right)\right]/e.m$$

where N_E_ is (CPS) specified line energy for samples, N_B_ is (CPS) specified line energy for background, e is the abundance of the gamma-peak in a radionuclide, η is the measured efficiency for specified gamma-peak energy, and m is mass of sample in (Kg) [11].

The uncertainty of activity u(A) was calculated using square limit equation as follow:-

(2) $$u\left(A\right)=A\sqrt{\sigma_{S}^{2}+\sigma_{\eta }^{2}+\sigma_{m}^{2}}$$

where $\sigma_{S}^{2}$ =[u(N_p_)/(N_p_)]^2^ for each sample, $\sigma_{\eta }^{2}$ =[u(η)/(η)]^2^ for the measured efficiency of each photo-peak energy, and $\sigma_{m}^{2}$ =[u(m)/(m)]^2^ for sample mass. The uncertainty of individual photo peak area is calculated assuming that a maximum inaccuracy of 2% due to contribution of other nuclides and it should be low average value. The total uncertainty of systematic and statistical efficiency was 5% [1].

## Results and discussion

### Radiometric analysis

**Fig. 2:**
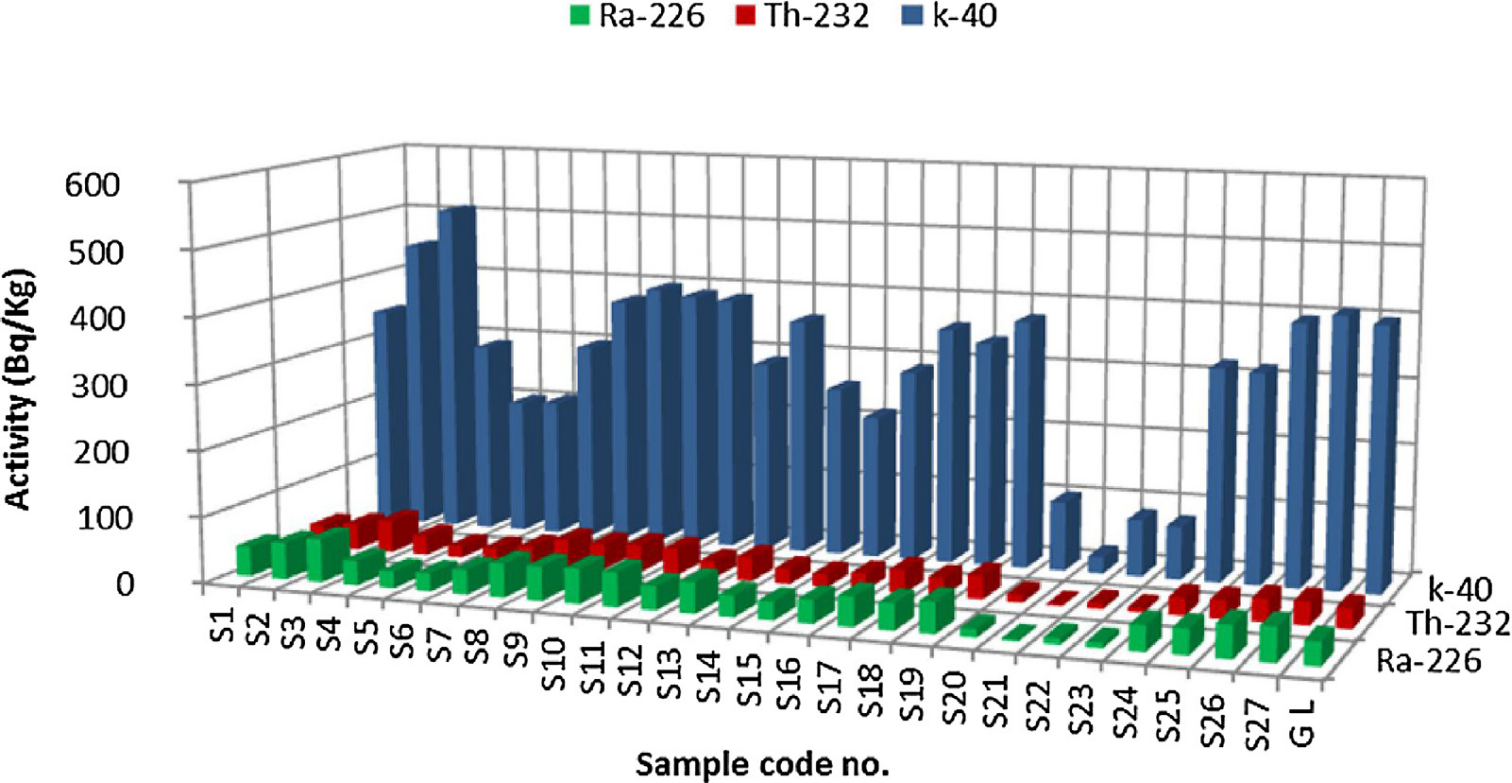
Activity concentrations of ^226^Ra, ^232^Th and ^40^ K (Bq/Kg) in different locations of Al Madinah, Saudi Arabia and last column represents the global limits.

In the current study, the measured activity concentrations of ^226^Ra, ^232^Th and ^40^K in 27 samples from different area of Harrat Al Madinah, Saudi Arabia are presented in Table 1 and showed in Fig. 2. The activity concentrations of ^226^Ra ranged from 3.05 ± 0.01 to 65.30 ± 0.14 Bq Kg^−1^ with average 37.54 ± 0.08 Bq Kg^−1^, the activity of ^232^Th ranged from 1.78 ± 0.04 to 49.06 ± 1.29 Bq Kg^−1^ with average 27.95 Bq Kg^−1^ and for ^40^K ranged from 25.05 ± 0.15 to 511.24 ± 2.42 Bq Kg^−1^ with average 300.64 ± 1.74 Bq Kg^−1^.

**Table 1:** Activity of Ra-226, Th-232 and K-40 (Bq/Kg), radium equivalent, the external and internal hazard index and level index.

Sample code no.	Type of sample	Activity (Bq/Kg)			Ra_eq_ (Bq/Kg)	Hex	Hin	Iγ
		Ra-226	Th-232	K-40				
S1	Stone: west harrat	45.51 ± 0.09	34.12 ± 0.57	341.84 ± 2.33	120.62	0.33	0.45	0.87
S2	Stone: west harrat	55.63 ± 0.12	39.15 ± 1.17	451.76 ± 2.25	146.40	0.40	0.55	1.06
S3	Stone: east harrat	65.30 ± 0.14	49.06 ± 1.29	511.24 ± 2.38	174.82	0.47	0.65	1.27
S4	Stone: east harrat	36.97 ± 0.09	29.66 ± 0.72	292.78 ± 2.40	101.92	0.28	0.38	0.74
S5	Stone: west harrat	25.60 ± 0.06	18.17 ± 0.23	205.49 ± 1.26	67.41	0.18	0.25	0.49
S6	Stone: west harrat	27.53 ± 0.06	19.19 ± 0.28	208.86 ± 1.02	71.05	0.19	0.27	0.51
S7	Stone: west harrat	36.90 ± 0.08	25.91 ± 0.50	301.54 ± 1.60	97.17	0.26	0.36	0.71
S8	Soil: west harrat	50.61 ± 0.10	40.46 ± 0.58	377.35 ± 1.84	137.52	0.37	0.51	0.99
S9	Soil: east harrat	51.00 ± 0.10	38.74 ± 0.73	400.24 ± 2.19	137.22	0.37	0.51	0.99
S10	Soil: west harrat	52.49 ± 0.10	41.52 ± 0.63	391.84 ± 2.37	142.03	0.38	0.53	1.03
S11	Soil: west harrat	51.45 ± 0.10	40.29 ± 0.59	387.82 ± 2.27	138.93	0.38	0.51	1.00
S12	Stone: west harrat	35.93 ± 0.08	25.45 ± 0.46	291.03 ± 1.81	94.74	0.26	0.35	0.69
S13	Soil: east harrat	46.50 ± 0.09	36.32 ± 0.55	360.91 ± 2.03	126.24	0.34	0.47	0.91
S14	Stone: west harrat	31.90 ± 0.07	22.81 ± 0.36	257.87 ± 1.53	84.37	0.23	0.31	0.61
S15	Stone: east harrat	27.71 ± 0.06	19.30 ± 0.28	217.43 ± 1.32	72.06	0.19	0.27	0.52
S16	Stone: west harrat	34.99 ± 0.08	25.06 ± 0.42	290.80 ± 1.75	93.22	0.25	0.35	0.68
S17	Stone: west harrat	44.51 ± 0.09	35.14 ± 0.52	360.31 ± 2.29	122.50	0.33	0.45	0.89
S18	Soil: east harrat	39.83 ± 0.09	27.67 ± 0.54	341.68 ± 2.01	105.70	0.29	0.39	0.77
S19	Soil: east harrat	46.36 ± 0.09	35.65 ± 0.52	378.49 ± 2.29	126.48	0.34	0.47	0.92
S20	Soil: west harrat	12.50 ± 0.03	11.59 ± 0.26	105.21 ± 0.64	37.18	0.10	0.13	0.27
S21	Stone: east harrat	3.05 ± 0.01	1.78 ± 0.04	25.05 ± 0.15	7.53	0.02	0.03	0.05
S22	Soil: east harrat	9.41 ± 0.03	8.61 ± 0.19	86.14 ± 0.51	28.35	0.08	0.10	0.21
S23	Soil: weast harrat	7.97 ± 0.02	6.03 ± 0.17	80.32 ± 0.48	22.77	0.06	0.08	0.17
S24	Stone: west harrat	37.39 ± 0.08	26.43 ± 0.47	323.64 ± 1.53	100.12	0.27	0.37	0.73
S25	Stone: west harrat	38.09 ± 0.08	27.10 ± 0.52	319.68 ± 1.91	101.46	0.27	0.38	0.74
S26	Soil: west harrat	48.14 ± 0.10	34.40 ± 0.69	396.93 ± 2.33	127.90	0.35	0.48	0.93
S27	Soil: west harrat	50.44 ± 0.11	35.11 ± 0.76	411.08 ± 2.42	132.30	0.36	0.49	0.96
average		37.54 ± 0.08	27.95 ± 0.52	300.64 ± 1.74	100.67	0.27	0.37	0.73
maximum		65.30 ± 0.14	49.06 ± 1.29	511.24 ± 2.42	174.82	0.47	0.65	1.27
minmum		3.05 ± 0.01	1.78 ± 0.04	25.05 ± 0.15	7.53	0.02	0.03	0.05

These results were compared with published global limits of ^226^Ra, ^232^Th and ^40^K by [15,16] which these values 35, 30 and 400 Bq Kg^−1^ respectively (Fig. 2).

Radium equivalent in Bq Kg^−1^ was calculated according to references of [11,12]. As shown in Table 1. We found that the Ra_eq_ varies between 7.53 and 174.82 Bq Kg^−1^ with average 100.67 Bq Kg^−1^ and the obtained results are lower than the global value 370 Bq Kg^−1^ which recommended by UNSCEAR [15].

**Fig. 3:**
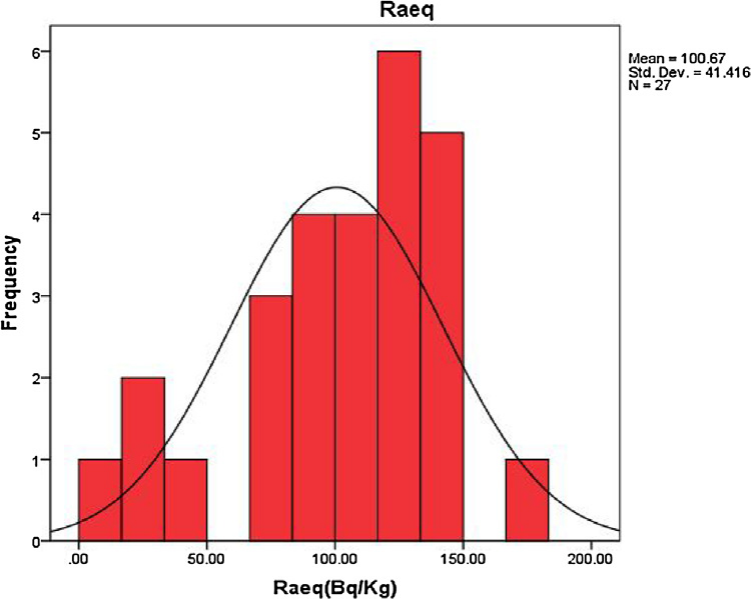
Frequency distribution of radium equivalent (Bq/Kg).

The frequency distribution curve of radium equivalent is plotted as shown in Fig. 3. That is clear that a nearly symmetric distribution with skewness equal -0.68, kurtosis equal -0.011and mean equal 100.67.

**Fig. 4:**
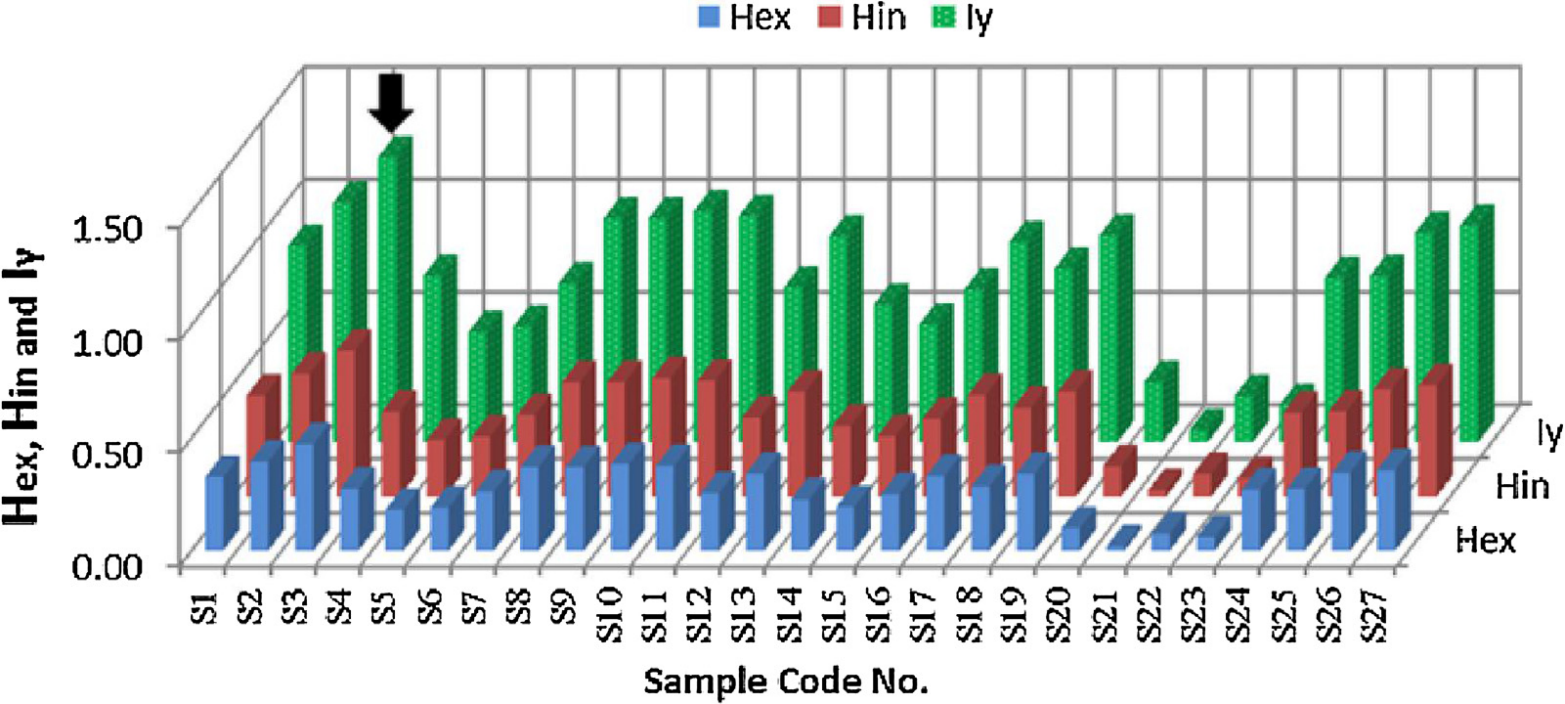
represents the external and internal hazard indices in addition to radiation level index.

The calculated external and internal hazard indices for each sample are presented in Table 1 using equation published by [[17], [18], [19], [20]]. H_ex_ ranged from 0.02 to 0.47 with average 0.27 and H_in_ ranged from 0.03 to 0.65 with average 0.37. It is clear that all samples indicated values less than unity as shown in Fig. 4. The values of radiation level index (I_γ_) were also shown in Table 1 and Fig. 4 using equation published by [21]. I_γ_ ranged from 0.05 to 1.27 with average 0.73. These results are close or less than unity except one sample that have sample code (S3) represented by black arrow in Fig. 4.

**Fig. 5:**
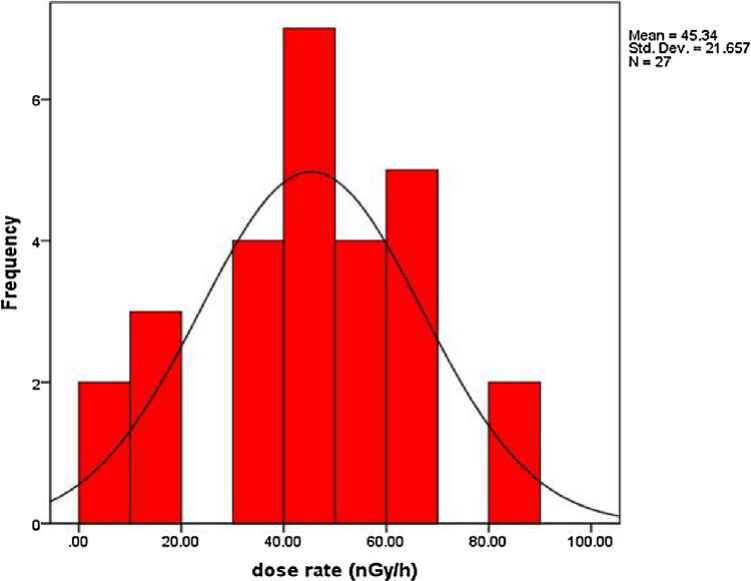
Frequency distribution of dose rate (nGy/h).

Table 2 represents the calculated radiation hazard parameters for investigated samples. According to the recent [22] the dose rates values lie within the worldwide range (18–93 nGy h^−1^) with average (55 nGy h^−1^). In the current work the dose rates lie between 3.53–81.12 with average 45.34 nGy h^−1^ which they are concordant with the worldwide ranges. Fig. 5 represents the frequency distribution curve of dose rate. As it is clear that a nearly symmetric distribution with skewness equal −0.457, kurtosis equal −0.404 and mean equal 45.34.

**Table 2:** Radiation hazard parameters for investigated samples.

Sample code no.	absorbed dose(nGy/h)	Eff Dose (outdoor) mSv/y	Eff Dose (indoor) mSv/y	Annual eff. Dose (μSv)	lifetime risk
S1	55.89	0.07	0.27	68.57	4.80
S2	68.19	0.08	0.33	83.66	5.86
S3	81.12	0.10	0.40	99.53	6.97
S4	47.20	0.06	0.23	57.92	4.05
S5	31.37	0.04	0.15	38.49	2.69
S6	33.02	0.04	0.16	40.51	2.84
S7	45.27	0.06	0.22	55.55	3.89
S8	63.55	0.08	0.31	77.98	5.46
S9	63.65	0.08	0.31	78.10	5.47
S10	65.66	0.08	0.32	80.57	5.64
S11	64.28	0.08	0.32	78.86	5.52
S12	44.11	0.05	0.22	54.12	3.79
S13	58.47	0.07	0.29	71.75	5.02
S14	39.27	0.05	0.19	48.18	3.37
S15	33.53	0.04	0.16	41.14	2.88
S16	43.43	0.05	0.21	53.28	3.73
S17	56.81	0.07	0.28	69.71	4.88
S18	49.36	0.06	0.24	60.56	4.24
S19	58.73	0.07	0.29	72.06	5.04
S20	17.16	0.02	0.08	21.06	1.47
S21	3.53	0.00	0.02	4.33	0.30
S22	13.14	0.02	0.06	16.12	1.13
S23	10.67	0.01	0.05	13.09	0.92
S24	81.12	0.10	0.40	99.53	6.97
S25	3.53	0.00	0.02	4.33	0.30
S26	45.54	0.06	0.22	55.88	3.91
S27	46.64	0.06	0.23	57.22	3.99
average	45.34	0.06	0.22	55.63	3.89
maximum	81.12	0.10	0.40	99.53	6.97
minmum	3.53	0.00	0.02	4.33	0.30

The calculated effective dose indoor and outdoor for all samples is less than unity. The worldwide average of annual effective dose is 70 μSv. Thus, most results are consistent with the global average except those recorded for S3 and S24 samples with relatively high values of 99.53 μSv.

Excess lifetime risk was calculated and noted in Table 2 using the following equation:-

(3) ELR = AEDE × D_L_ × R_F_

where AEDE is annual effective dose equivalent, D_L_ is duration of life (70 year) and R_F_ is risk factor (Sv^−1^). Fatal cancer risk per Sievert for stochastic effects, ICRP 60 uses the magnitude of 0.05 for the public [23,24]. The calculated lifetime risk ranges from 0.30 to 6.97 with average 3.89.

Table 3 lists the comparison of mean radioactivity concentrations of ^226^Ra, ^232^Th and ^40^K in the present study and other locations in different countries in the world. In some countries in Asia, the mean activity concentrations range from 12.53 to 70 for ^226^Ra, 10.5 to 64.9 for ^232^Th and 138.1 to 436.1 ^40^K. In some countries in Europe, the mean activity concentrations range from 25.2 to 37 for ^226^Ra, 28.9 to 40 for ^232^Th and 384.4 to 667 ^40^K. In some countries in Africa, the mean activity concentrations range from 12.24 to 46.1 for ^226^Ra, 8.46 to 65.73 for ^232^Th and 136.3 to 412.5 ^40^K. It is clear that the results of different continents are in the Global permitted ranges which published with [8,15] except India.

**Table 3:** Comparison of mean radioactivity concentrations in Soil between the present study and other locations in the world.

Continent	country	Ra-226 (Bq/Kg)	Th-232 (Bq/Kg)	K-40 (Bq/Kg)	Reference
Asia	Saudi Arabia	37.5	28	300.6	Present study
	Thailand	60.5	64.9	431.8	[27].
	Kuwait	12.53	10.65	300	[28].
	Jordan	57.7	18.1	138.1	[29].
	India	70.0	34.8	436.1	[30].
Europe	Spain	25.2	28.9	384.4	[31].
	Serbia	33.2	49.1	379	[32].
	Turkey	37	40	667	[2].
Africa	Egypt	12.24	8.46	136.3	[33].
	Cameroon	46.16	65.73	215.91	[34].
	South Africa		47.65	87.17	[24].
	Nigeria	41	29.7	412.5	[35].
America	Brazil	---	5.32	34.15	[36].
Global limit (mean)		17-60 (35)	11-68 (30)	140-850 (400)	[8].

## Chemical analysis using XRF

Oxides of major elements of studied samples were carried out using EDXRF (ARL Quant’X manufactured by Thermo Fisher Scientific Seller, USA) and listed in Table 4. There is a strong correlation between rich uranium content and chemical composition especially iron and manganese oxides due to their high ability to absorb uranium [25,26]. The content of Fe_2_O_3_ ranges from 5.5% to 13.6% with average 9% and the content of MgO range from 5% to 8.3% with average 6.2%. All element contents are concordant with the obtained results by [26].

**Table 4:** Metal Content in soil samples using XRF.

Sample code	Al_2_O_3_	CaO	Fe_2_O_3_	K_2_O	MgO	MnO	Na_2_O	SiO_2_	TiO_2_
S1	17.0	11.5	7.5	1.5	7.5	0.2	^*^LLD	53.6	1.2
S2	19.6	8.8	12.8	0.6	5.1	0.3	LLD	49.7	2.8
S3	17.4	11.4	6.8	1.6	6.4	0.1	LLD	54.5	1.1
S4	16.9	11.7	6.8	1.7	6.2	0.1	LLD	55.6	0.8
S5	18.3	8.9	13.4	0.5	6.7	0.3	LLD	48.5	3.0
S6	12.2	4.3	5.7	0.3	8.3	0.1	43.0	24.6	1.3
S7	21.8	8.4	9.7	0.5	6.3	0.2	LLD	51.3	1.5
S8	18.5	7.5	6.9	1.8	5.5	0.1	LLD	58.7	0.8
S9	17.1	11.4	6.8	1.6	6.4	0.1	LLD	55.5	0.9
S10	18.1	7.4	6.7	1.7	5.0	0.1	LLD	59.9	0.9
S11	18.4	7.3	6.8	1.7	5.9	0.1	LLD	58.4	1.3
S12	18.7	8.5	13.1	0.7	5.5	0.3	LLD	49.8	3.0
S13	17.0	11.7	6.6	1.6	6.7	0.1	LLD	55.3	0.9
S14	18.6	8.5	13.6	0.8	5.0	0.3	LLD	49.9	3.1
S15	22.2	8.2	9.6	0.4	6.1	0.2	LLD	51.5	1.4
S16	18.7	8.3	13.0	0.8	5.3	0.3	LLD	50.3	3.1
S17	16.8	11.8	7.0	1.7	6.3	0.1	LLD	55.3	0.8
S18	17.9	8.1	13.2	0.7	5.2	0.2	LLD	51.3	3.1
S19	15.3	12.4	7.4	1.7	7.0	0.1	LLD	54.7	1.2
S20	18.2	7.4	6.6	1.7	5.8	0.1	LLD	59.2	0.8
S21	20.8	8.8	10.8	0.4	6.9	0.2	LLD	49.8	2.0
S22	16.7	11.7	6.8	1.7	6.2	0.1	LLD	54.9	1.2
S23	17.2	8.0	10.6	1.3	6.1	0.2	LLD	54.0	2.4
S24	11.5	3.4	5.5	0.6	7.7	0.1	44.2	25.8	1.3
S25	18.3	8.5	13.5	0.7	5.3	0.3	LLD	50.0	3.2
S26	12.5	4.3	5.7	0.3	7.9	0.1	42.7	25.0	1.4
S27	17.3	7.6	11.5	1.3	5.9	0.2	LLD	53.4	2.7
average	17.5	8.7	9.0	1.1	6.2	0.2	43.3	50.4	1.8
maximum	22.2	12.4	13.6	1.8	8.3	0.3	44.2	59.9	3.2
minimum	11.5	3.4	5.5	0.3	5.0	0.1	42.7	24.6	0.8

LLD: Lower Limit of Detection.

## Conclusions

Radiometric and chemical analysis was carried out in soil and stone samples collected from Harrats Al Madinah in western region of the kingdom of Saudi Arabia. The contribution of radionuclides in Al-Madina city represents 37% for ^226^Ra, 36% for ^232^Th and 27% for ^40^K. The results of the current study are within the global allowable limits, so this area of samples is safe for human beings that they live in. When we compared the content of elements in this study with other countries in different continents, we found that the concentrations of elements agree with them. Some of these elements have strong ability to sorption uranium, thorium and potassium. The current study is considered as the first baseline reference data about the natural radionuclides and elemental contents in the area of the study. The researchers recommend that follow-up of the study area should be raised to record the changes and develop a pollution control strategy.
